# Use of Moxa in Rheumatism

**Published:** 1842-10

**Authors:** John Leney


*Use of Moxa in Rheumatism.* By John Leney.—The laboring classes in Ireland, from exposure and insufficient clothing, are very aubject to local rheumatism. Rheumatic fever is a disease comparatively rare among them. Those forms of most frequent and distressing occurrence are lumbago, rheumatism of the deltoid muscles, and sciatica. In obstinate cases of local rheumatism I invariably apply moxa; there is no application so immediately successful, or which creates less inconvenience to the patient, especially if he be a laborer. At dispensary my ordinary treatment for lumbago is castor oil and turpentine draught, as follows: ℞. Olei ricini, ℥j.; terebinth. syrupi, aa ʒiij.; aq. menth. piperit. ℥j M. Ft. haustus.

The turpentine epithem on flannel to the loins, kept on from twenty minutes to one hour, or until, in fact, the patient exclaims loudly for its removal; it often brings out very minute vesicles, and the cuticle desquamates. Also ten or fifteen grains of Dover's powder at bed-time, the feet and legs bathed in warm water.

In rhematism of the deltoid muscles, and sciatica, apply moxa by all means, as blisters and tartar-emetic ointment disable the patient, and render him incapable of working. The laboring classes cannot afford to be confined to the house, whereas after the application of moxa the patient may return to his work at once. I shall instance a few aggravated cases in which it proved successful.

James Pender, ætat. 56 years, got sciatica from standing in water, the principal part of a day, washing sheep. He had been confined to the house, nay, to the bed, for six weeks, and had used a variety of means—stupes, blisters, warm plasters, &c., without deriving the smallest benefit. I applied moxa in three places; first, at the origin of the nerve, each application three inches distant, and following the course ot the nerve down the limb. This man had eighteen applications. From the first burning the disease descended, so that I had to continue it down the limb to the ankle. Several of the burns continued open for some time. One particularly down the calf of the leg discharged pus for three weeks. This man has been ever since (now five years) at labor without having had any return of the disease.

Judy Cavanah, æt. 57 years, confined to bed six weeks from sciatica of right limb: was burned in four places, on those parts in the course of the nerve which she indicated as most painful. She was immediately cured. That was three years ago. Last winter she had the disease in the left limb, was confined to bed, and suffered excruciating pain. The moxa again relieved her, being applied in seven places. She had not any internal medicine during either attack.

Bridget Doyle, æt. 48 years, living at a distance of three miles, being informed of her sister's recovery, was brought to the dispensary on a car, and carried in, being unable to put the limb under her; had been confined to bed for fifteen weeks. This woman was burned in three places; and so immediate was her recovery, that she walked home with her husband in two days after. He, laboring under rheumatism of the deltoid muscle of the right arm, which rendered him incapable of working (having been employed in threshing corn), was burned in two or three places, which enabled him to return to his work the following day.

A stout, able-bodied young man, who had undergone a variety of treatment, was blooded, blistered, purged, used sudorifics, and finally had tartar-emetic ointment rubbed in throughout the whole limb, from which he had no relief; from this unlimited application of tartar-emetic ointment, a mass of tubercular swellings remained in the skin, so thick that it was difficult to procure places for the application of moxa; he was burned in three places, and became immediately well, now two years since. He has had no return. I selected the foregoing cases to show its permanent benefit.

June 15th, 1842.—Jane Gaskin, æt. 14 years, has had sciatica of the left limb for five weeks, and been confined to the house from pain and inability to move the limb (the limb is smaller than the opposite). Moxa applied in three places, from the ischium to popliteal space; lost all pain immediately, and was able to return at once to her ordinary avocations. This girl had been blistered, used tartar-emetic ointment, aperients, &c., without any benefit; has had no return of pain up to this day (June 30th).

My experience would lead me to say that if there be not an immediate relief obtained on the first or second application, the case will be tedious, or pass down the limb, as in Pender's case. I am at present treating a case exactly like Pender's, in which the pain has left the parts first burned, and is now taking the course of the posterior tibial nerve.

The way in which I make moxa, and apply it, is the following:—In a strong solution of nitrate of potass, soak a piece of lint, then dry it, cut off pieces the size of the thumb-nail, fasten them at one extremity over the seat of pain by a little adhesive plaster, set fire to the opposite extremity, then blow on it strongly with a blow-pipe. The pain during the operation is very severe, causing an immediate puckering of the cuticle. Those cases did best, in which, under its influence, a general perspiration broke out.

Had I not mislaid my note-book, I should have been able to have detailed these cases more accurately, and given some cases of affections of the knee and spine in which it proved successful. For the last six years I have been using moxa with undeviating success, and cannot instance a case in which it failed. When first proposed, the idea of being burned was such that it prevented many submitting to it; but now that they have had demonstrative proof of its efficacy, they gladly submit, preferring it much to the application of blisters.—*London Med. Gazette.*

